# Treatment of congenital short palate using bilateral buccal musculomucosal flaps

**DOI:** 10.1080/23320885.2020.1756821

**Published:** 2020-05-06

**Authors:** Shinji Kobayashi, Yukie Ohashi, Ryouko Fukushima, Takashi Hirakawa, Toshihiko Fukawa, Toshihiko Satake, Jiro Maegawa

**Affiliations:** a Department of Plastic and Reconstructive Surgery, Kanagawa Children’s Medical Center, Yokohama, Japan; b Department of Speech treatment, Kanagawa Children's Medical Center, Yokohama, Japan; c Hirakawa Orthodontic Clinic, Yokohama, Japan; d Fukawa Orthodontic Office, Yokohama, Japan; e Department of Plastic and Reconstructive Surgery, Yokohama City University Hospital, Yokohama, Japan

**Keywords:** Congenital short palate, buccal musculomucosal flap, Levator veli palatini muscle, Randall classification, velopharyngeal insufficiency

## Abstract

A case of congenital short palate was treated by bilateral buccal musculomucosal flaps. The levator veli palatini muscle formed a continuous sling, but the anterior portion was attached to the posterior border of the hard palate. The speech outcome improved from severe to normal.

## Background

The causes of velopharyngeal insufficiency (VPI), excluding neurogenic and myogenic disorders, can include anatomical abnormalities. Most VPI patients without the stigmata of a submucous cleft palate (SMCP) might be identified as having congenital short palate (CSP), which has a continuous levator veli palatini muscle (LVPM) without separation, or occult cleft palate (OCP), which has a separation of the LVPM [1–6].

The findings should be evaluated comprehensively by a combination of speech assessment, nasopharyngoscopy (NPS), lateral pharyngography (LPG), multiple videofluoroscopy and/or MRI. [7–10]. It is important to examine not only the nasopharynx, but also the length and movement of the soft palate. The purpose of this case report is to demonstrate the reconstruction of the soft palate using a buccal musculomucosal flap (BMMF) [11–16] for the treatment of CSP (Videos 1 and 2).

## Case

**Figure 1.**
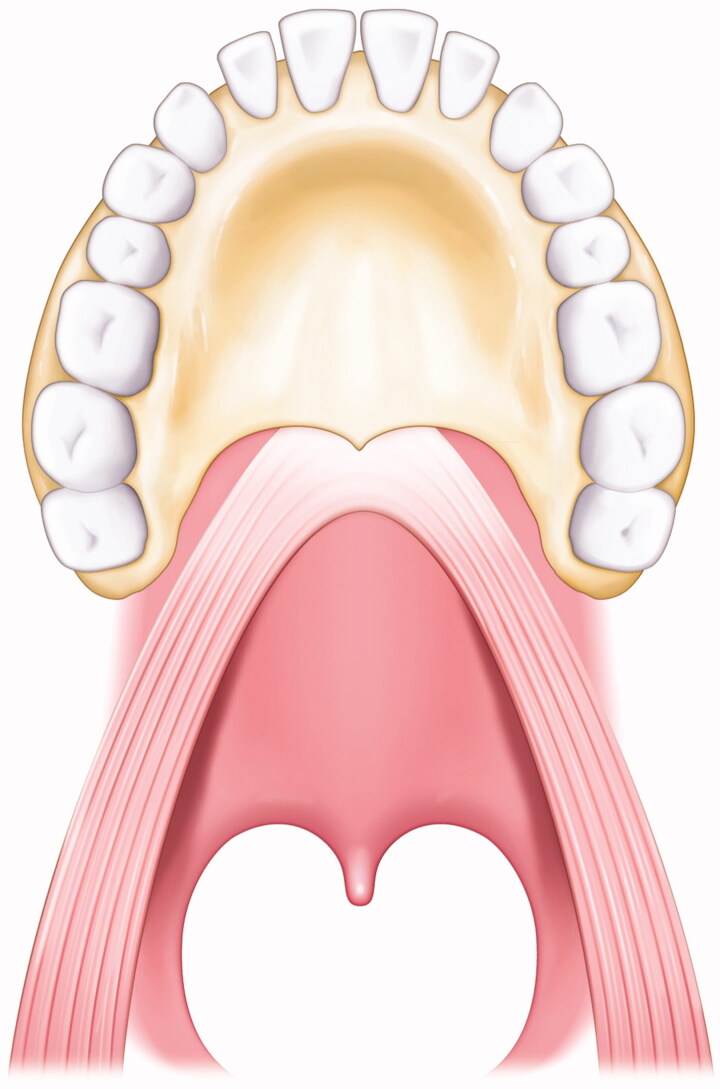
Illustration of the state of the LVPM. The LVPM continues from side to side, and the anterior portion is attached to the posterior border of the hard palate.

**Figure 2.**
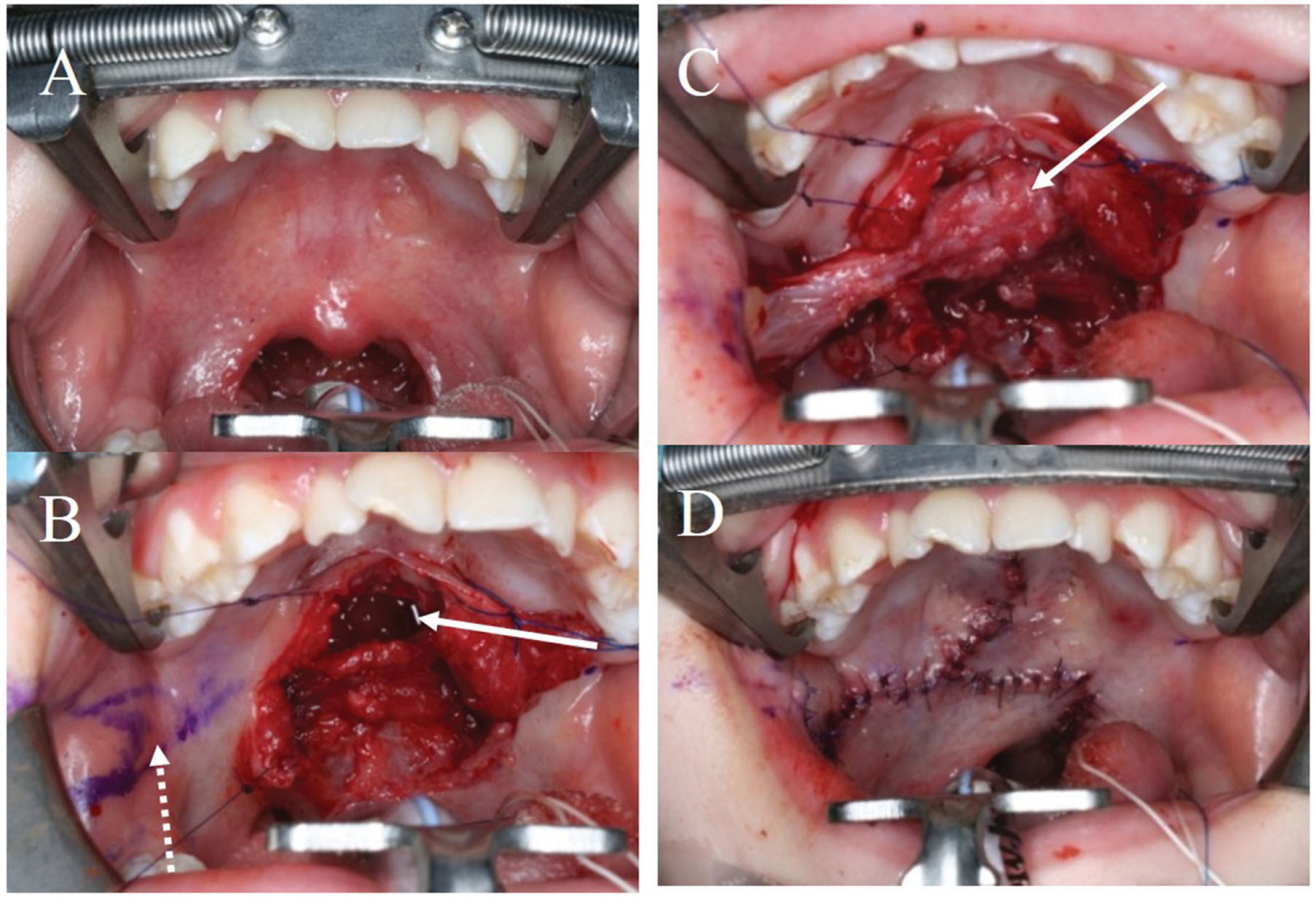
The first operation. (A) Intraoral findings. There are no features of SMCP. (B) Separation of the abnormal attachment of the LVPM from the PNS and soft palate elongation on the nasal side. A space occurs after posterior elongation (white arrow). The pedicle of the BMMF is denuded (white dotted arrow). (C) The space is filled by the right BMMF (white arrow). (D) After the first operation.

**Figure 3.**
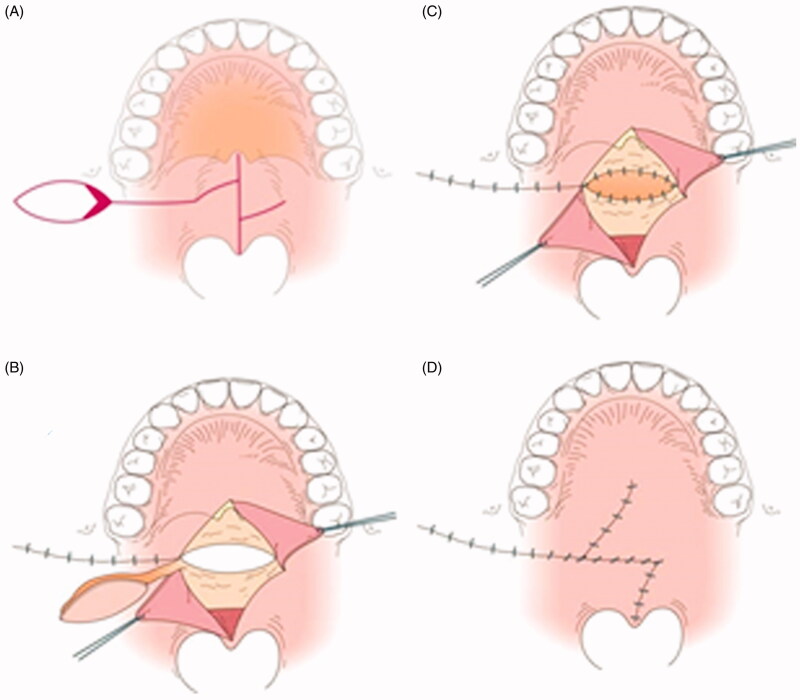
Illustration of Figure 2(A–D). A: The pedicle of the right BMMF is denuded (red area).

**Figure 4.**
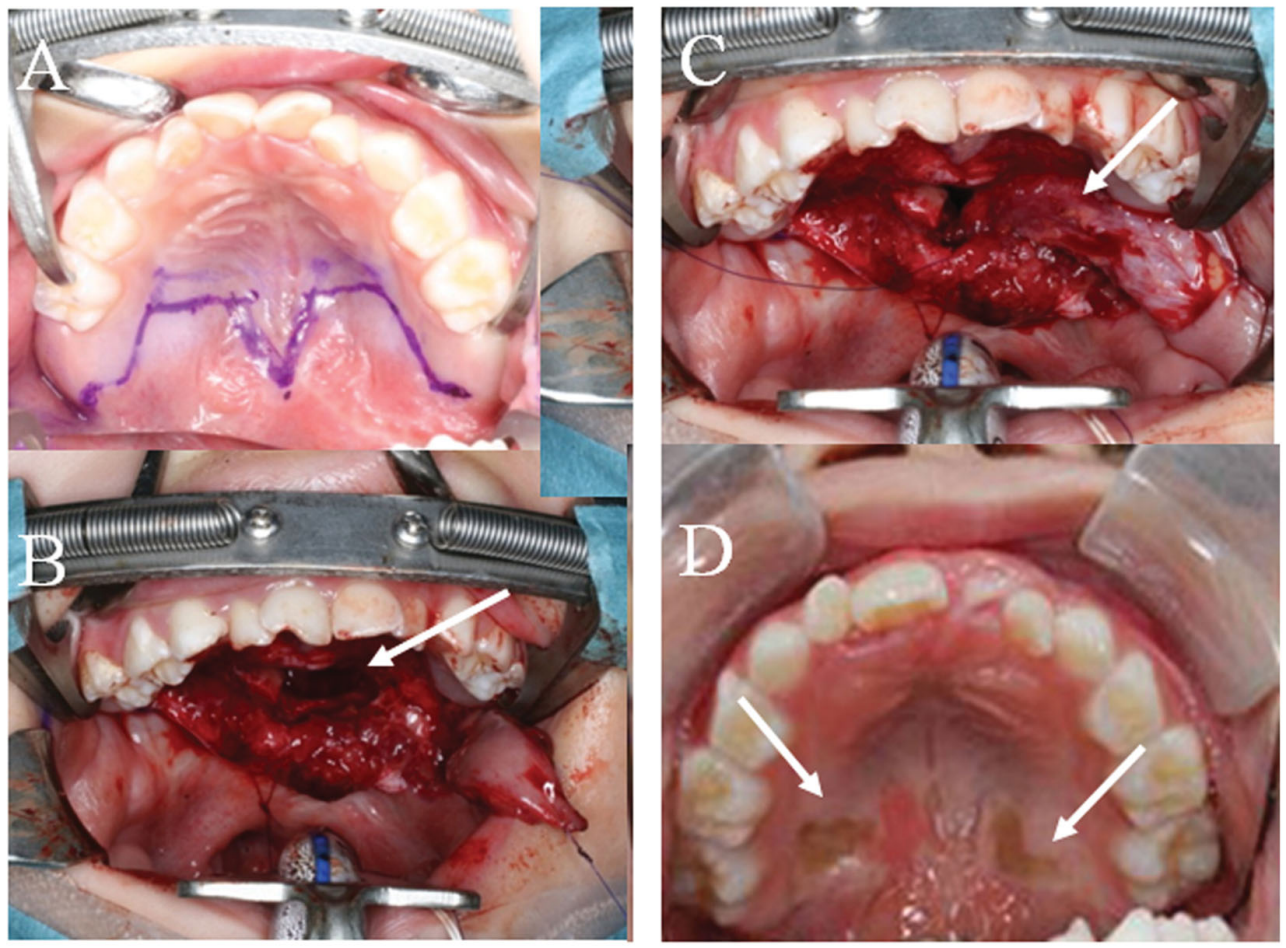
The second operation. (A) Design of the second operation. A left BMMF for soft palate elongation on the nasal side and a skin graft (SG) for soft palate elongation of the oral side were designed. (B) Soft palate elongation on the nasal side. A space occurs after posterior elongation (white arrow). (C) The space is filled by the left BMMF (white arrow). (D) One year after the second operation. Engrafted SGs (white arrow).

**Figure 5.**
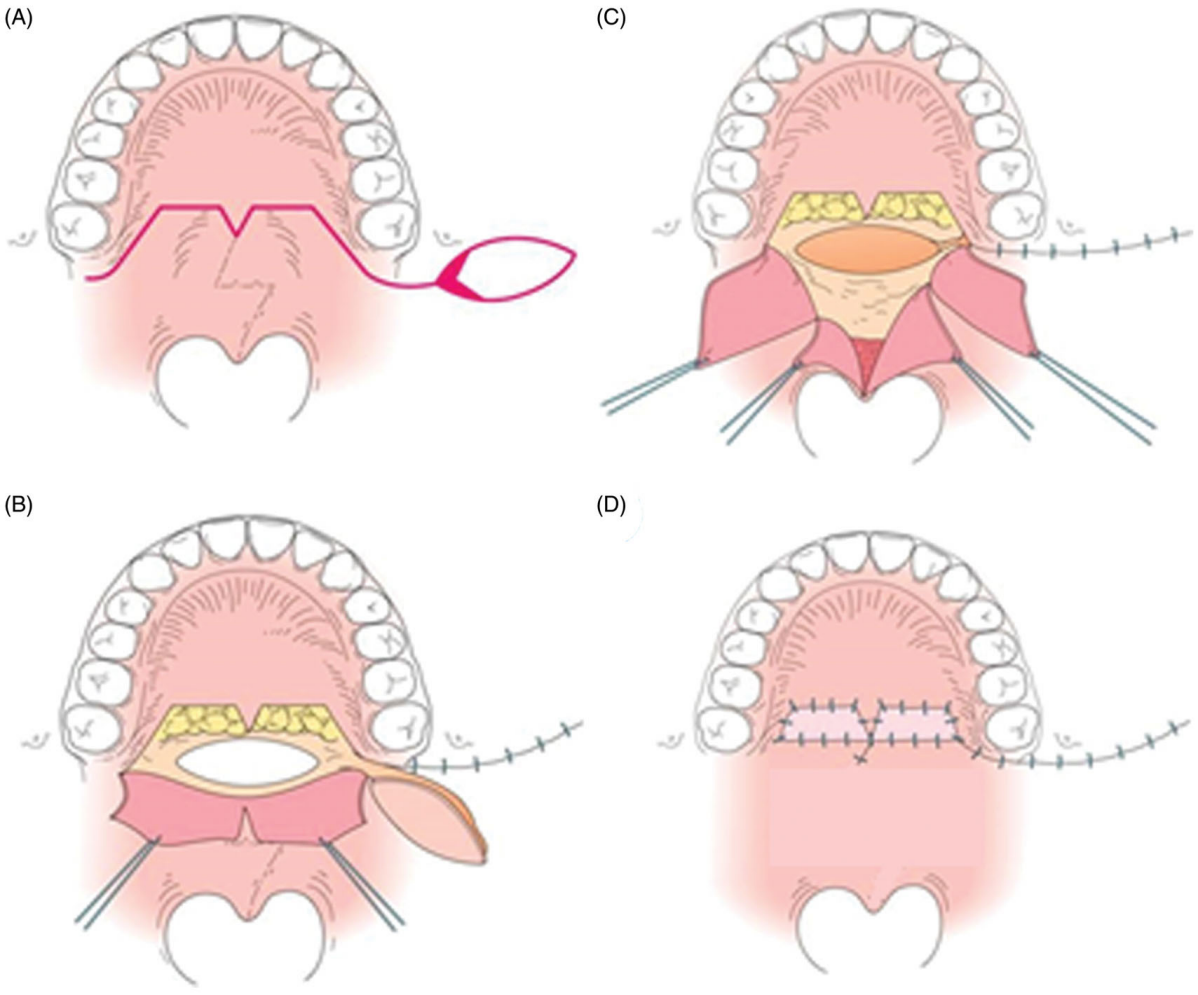
Illustration of Figure 4(A–D). A: The basal portion of the left BMMF is denuded (red area).

**Figure 6.**
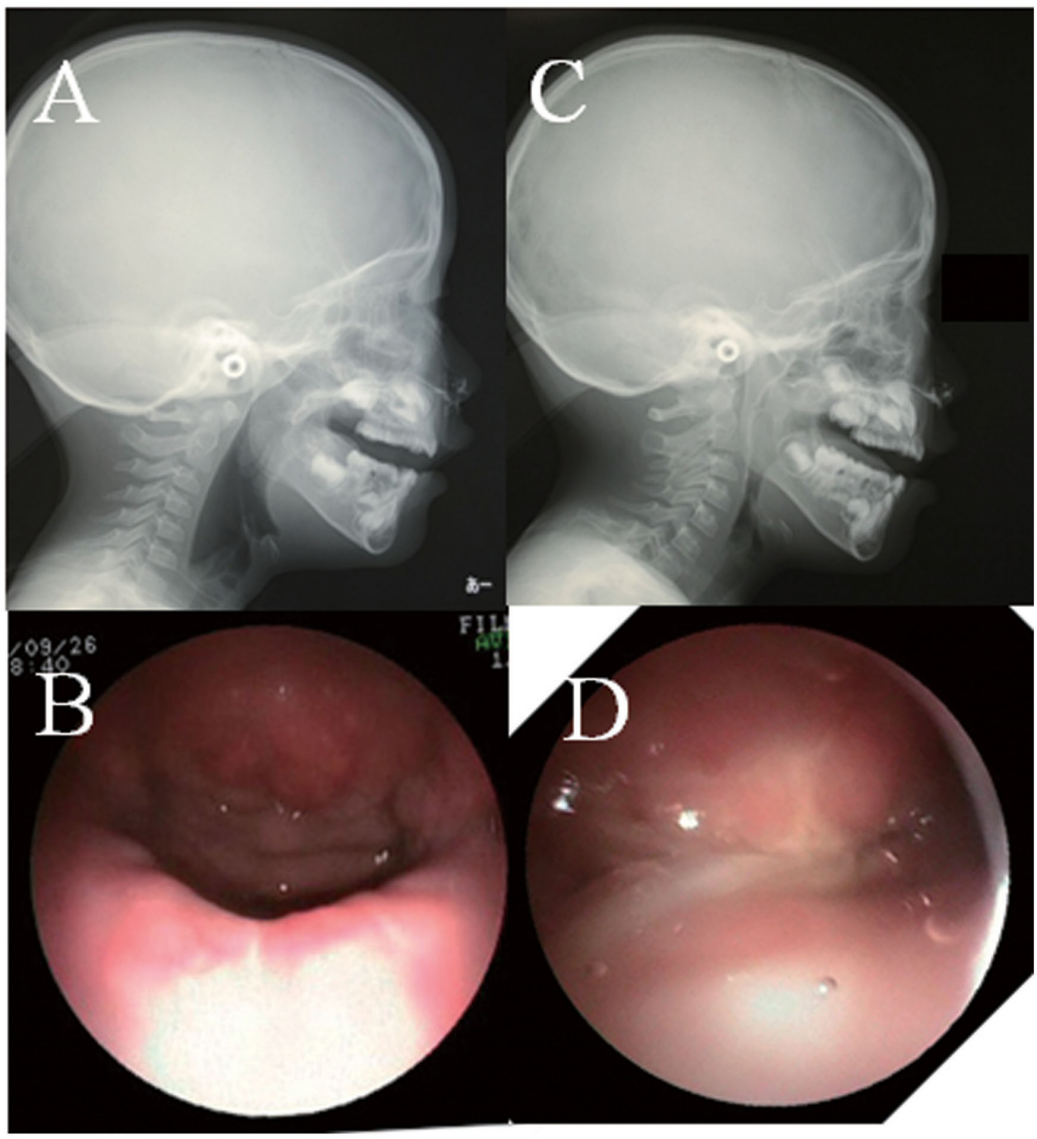
Findings of NPS and LPG. (A) LPG during phonation before the first operation. No contact between the posterior pharynx and soft palate (black arrow). (B) NPS midline view of the velopharyngeal valve during phonation before the first operation. A large defect is seen. (C) LPG during phonation after the second operation. The space between the posterior pharynx and the soft palate is completely closed (black arrow). (D) NPS midline view of the velopharyngeal valve during phonation after the second operation. The space between the posterior pharynx and the soft palate is completely closed.

A 4-year-old girl was noted to have CSP without intellectual disability, and she seemed to have a short palate and a deep pharynx. On assessment using the University of Pittsburgh weighted values for speech symptoms associated with VPI, her speech outcome was: nasal emission, 5/5; facial grimace, no; hypernasality, 3/4; phonation, N.A; and articulation, 6/10 [17,18]. NPS and LPG were performed preoperatively to evaluate the movement of the soft palate and the lateral wall of the pharynx, as well as the anatomical structure of the palatopharynx. There was no contact between the posterior pharynx and soft palate during phonation, and a large defect was seen on NSP (Supplementary Video 1). Furthermore, her soft palate length was classified as Randall classification type III at the beginning of the operation [19]. As for intraoperative findings, her LVPM continued from side to side completely, as in the normal anatomy, and the anterior portion was attached to the posterior border of the hard palate (Figure 1). Therefore, Furlow’s palatoplasty and intravelar veloplasty, which separate the LVPM from the nasal mucosa, were not performed to maintain the continuity of the LVPM, and for the first operation, a right BMMF (35 mm × 12 mm), of which the pedicle was denuded, was performed for soft palate elongation on the nasal side, along with Z-plasty without LVPM separation on the oral side (Figures 2 and 3). The operative time was 3 h, and the hospital stay was 8 days. After the first operation, her speech outcome improved from severe to moderate, but her VPI remained. For the second operation, oral mucosal flaps were raised because the soft palate already had scar formation from the previous Z-plasty, and the LVPM remained without separation again, because the LVPM was confirmed to be completely continuous. Therefore, a left BMMF (35 mm × 12 mm) for soft palate elongation on the nasal side was performed again, and a skin graft, taken from her groin on the oral side, was performed for the defect of the hard palate (Figures 4 and 5). The defect of the nasal surface was closed during phonation on NPS after the second operation. She was fed through a gastric tube for 7 days to prevent misalignment of the dressing on the graft due to food in the mouth, and over the next 7 days, the food was changed from a light diet to a regular diet. Her postoperative speech outcome improved (nasal emission, 0/5; facial grimace, no; nasality, 0/4; phonation, 0/3; and articulation, 0/10) after two operations. There was also no space between the posterior pharynx and the soft palate during phonation on LPG (Figure 6, Supplementary Video 2).

## Discussion

The treatment of CSP patients is difficult and controversial. Our strategy for the treatment of CSP patients with a short soft palate and continuity of the LVPM is to elongate the soft palate using bilateral BMMFs as the first choice. We believe that the anatomical abnormalities should be repaired as much as possible to the normal state.

This procedure had three advantages. The first advantage was that the oral cavity could be kept in the normal state without narrowing of the cavity by a pharyngeal flap. Therefore, this procedure did not cause postoperative sleep apnea that might sometimes occur with a pharyngeal flap [20–22].

The second advantage was that the continuous LVPM could be saved without separation to elongate the soft palate. It was considered that BMMF without separating the continuous LVPM would be preferred to Furlow’s palatoplasty with separation of the muscle, because this case had a completely normal LVPM with a short palate. It was also considered that the extended effect of Furlow’s palatoplasty for the short soft palate might be less than that of a BMMF.

On the other hand, Nguyen and colleague reports that progressive tightening of the LVPM improves velopharyngeal dysfunction [23]. Therefore, it might be better to divide the muscle and resuture under some tension overlapping the muscular stumps, though there is no evidence that muscle activity would change by separating it from the mucosa for CSP with the continuous LVPM

The third advantage was that subsequent pedicle management was not needed, because the denuded pedicle of the BMMF was buried under nasal mucosa. If the pedicle of the BMMF was not denuded, the mucosa of the pedicle or the pedicle itself might need another operation to remove it later.

Meanwhile, there were two disadvantages of this procedure. The first disadvantage was that the procedure required two stages. Some reports have stated that a combination of multiple techniques such as intravelar veloplasty with a pharyngeal flap or Furlow’s palatoplasty with sphincter pharyngoplasty at single-stage surgery would increase the possibility of achieving a good speech outcome [24–26]. The multiple techniques that combined a pharyngeal flap or sphincter pharyngoplasty might be excessive for some patients who would have a good result following the first BMMF at the first operation. The second disadvantage was that the second operation took 3.5 h, and a hospital stay of 14 days was needed to check the graft. This complicated procedure with a BMMF and grafting requires a longer operation and hospital stay than other procedures [24–26]. Nevertheless, we should have a hospital stay of 7 days, because 14 days to check the graft seemed a long time. Furthermore, if the speech outcome does not improve after our procedure, retropharyngeal augmentation by cartilage [27] and fat [28–30] would be considered.

## Conclusion

A case of CSP was treated by bilateral BMMFs to elongate the soft palate. The patient’s speech outcome improved from severe to normal.

## Supplementary Material

Supplemental Material

Supplemental Material
